# “Well, what we can do is […] to organize data, to evaluate studies”—Self-images of public health academics in Germany during the COVID-19 pandemic: a qualitative study

**DOI:** 10.1186/s12889-024-19167-5

**Published:** 2024-06-24

**Authors:** Julia Piel, Julian Prugger, Anne Meuche, Marilena von Köppen, Tizia Rosendorfer, Christian Apfelbacher

**Affiliations:** 1 Institute of Social Medicine and Health Systems Research, Otto von Guericke University, Magdeburg, Germany; 2 Munich School of Philosophy, Munich, Germany

**Keywords:** Public health academia, Self-image, COVID-19 pandemic, Germany, Crisis management

## Abstract

**Background:**

Despite the significant role of scientific knowledge pertaining to public health, the discipline of public health remained outside the centre stage within the pandemic discourse. Against this background, we investigated the role of German public health academics during the pandemic in our study, focusing on their orientations and associated values.

**Methods:**

We interviewed 21 public health scholars from Germany and collected 36 documents published by public health scientific societies. We analyzed data by grounded theory and situational mapping.

**Results:**

We identified five types of self-images identified among healthcare academics: the scientific study supplier, the expert facing political issues, the restrained scholar, the public informer and the changemaker. The typology yields insights into the multiple dimensions of public health and its role in times of crisis.

**Conclusions:**

The findings provide implications to inter- and transdisciplinary interaction and to managing the expectations of public health professionals in relation to crisis management.

## Introduction

Throughout the Covid-19 pandemic, scientific expertise was called for to address the complex societal challenges arising from the uncertainty and dynamics of the situation. Scientific knowledge was sought after as a foundation for political decision-making [14, 16]. Evidence-based policy was partly taken for granted as the key to successful pandemic management [19]. In Germany and other countries, public health (PH) did not appear to be central in the pandemic discourse. PH as one of many disciplines however introduces different perspectives on the conditions of health in societies. Further, it provides a holistic view of PH emergencies such as the COVID-19 pandemic [1, 6].

Like most scientific disciplines, the PH discipline endorses a certain set of values that are most prominently articulated by the World Health Organization in the principles of the Ottawa Charter for health promotion: building health public policy, creating supportive environments for health, strengthening community action for health, developing personal skills and re-orienting health services with overarching goals to achieve ‘health for all’ and ‘health in all policies’ [34–36]. Given these wide-ranging values and the notion of PH as both ‘art’ and ‘science’, ‘theory’ and ‘practice’, the roles that PH academics take on are manifold. During the COVID-19 pandemic they ranged from epidemiological research to the transmission dynamics of SARS CoV-2 and politically engaged PH scholarship [15].

Furthermore, PH is best understood in terms of a heterogeneous domain. The normative nature of PH arguably forces PH scientists to consider their implicit normative sets of beliefs and values. As Schnabel et al. convincingly argue, the scientist‘s world(view) is partially fabricated through beliefs and values about the social and natural world [29]. Following Schnabel, we locate normative beliefs and values within the PH academics’ self-images and perceptions of their role as academics. The pandemic naturally put self-images and role-perceptions to the test. In contrast to other disciplines, its representatives working in different fields internalized different understandings of their profession due to heterogeneous tasks, roles and values. These values became challenged during the COVID-19 pandemic [2, 13]. PH experts in different scientific fields held divergent opinions on the policy measures implemented to mitigate the pandemic. There appeared to be no unified self-image among PH professionals based on shared values. However, the COVID-19 pandemic made PH as a discipline visible because it provoked a PH response, through policy documents, opinion papers, advisory roles or research activities. This setup creates a unique opportunity to examine self-images in PH. Changes in values and self-images or expression during the pandemic have indeed been examined in other groups, for instance, in adult people from Poland or Chinese nurses [5, 7, 31]. In PH academia, however, self-images remain unexplored.

## Research interest

We investigated self-images among the heterogeneous German PH community in dealing with the crisis. First, we were interested in how academics experienced their own role during the pandemic facing political pandemic management (1). Second, we asked which tasks and values they associated with the self-image of their discipline in times of crisis (2).

## Material and methods

Since research and scientific expertise were ‘in demand’ during the pandemic, we focused our qualitative study on academia. To foster the credibility and trustworthiness of the data acquired, we followed the criteria of qualitative research regarding the research team, reflexivity, the study design, analysis and findings. The research team consisted of two female research fellows experienced in qualitative research with master’s degrees in public health and social sciences and a male professor of epidemiology and health systems research. The two female researchers conducted the interviews and coding. Interviewees did not know the interviewers beforehand and were interviewed after a short introduction and some information about the research project. The interviews, the coding and the situational analysis (SA) were repeatedly discussed among the team. A detailed account of all other information including the study design can be found in our published study protocol as well as in the discussion section [28].

**Fig. 1:**
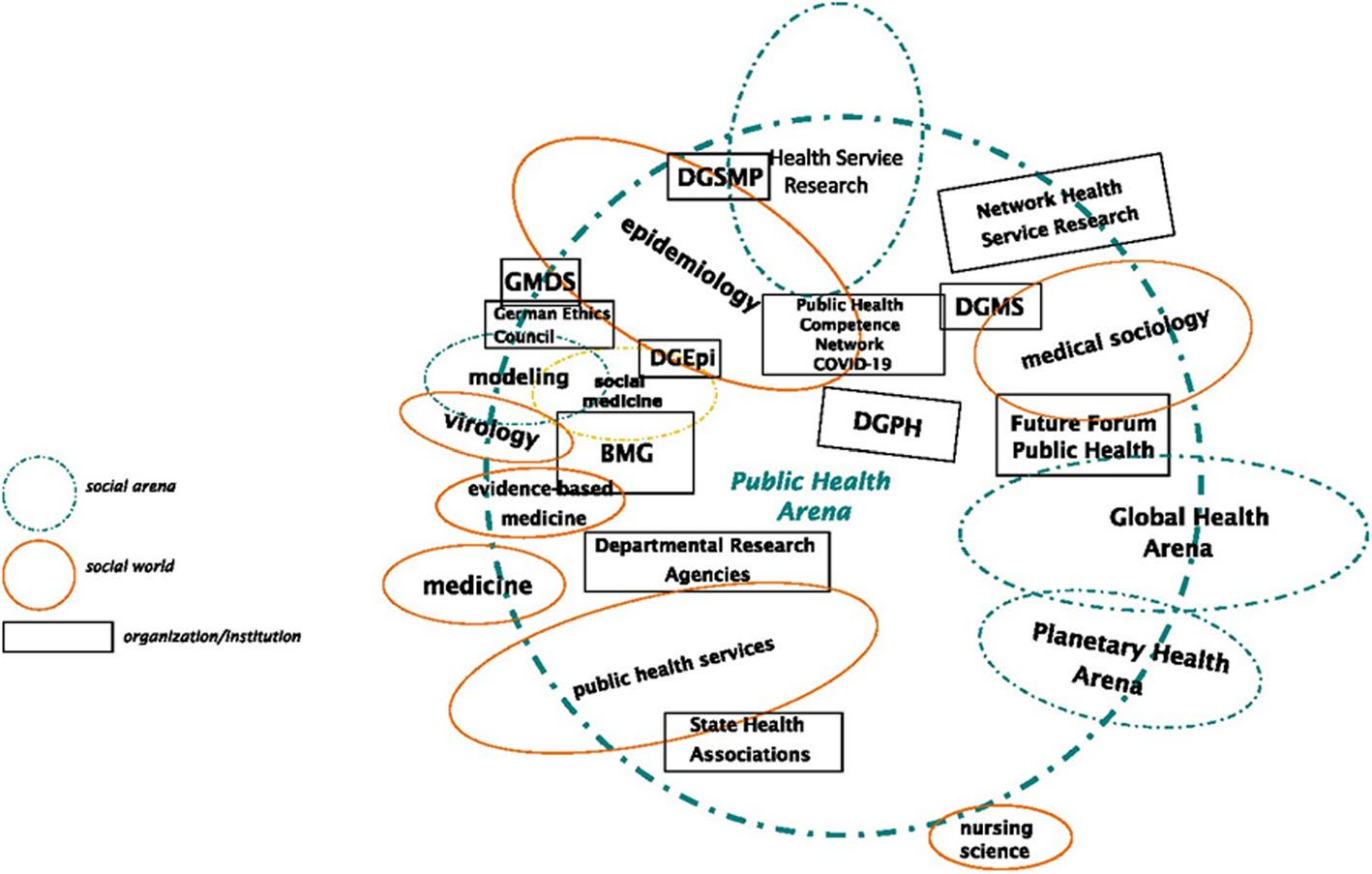
Social arena PH. Abbreviation: BMG: Bundesministerium für Gesundheit

**Fig. 2:**
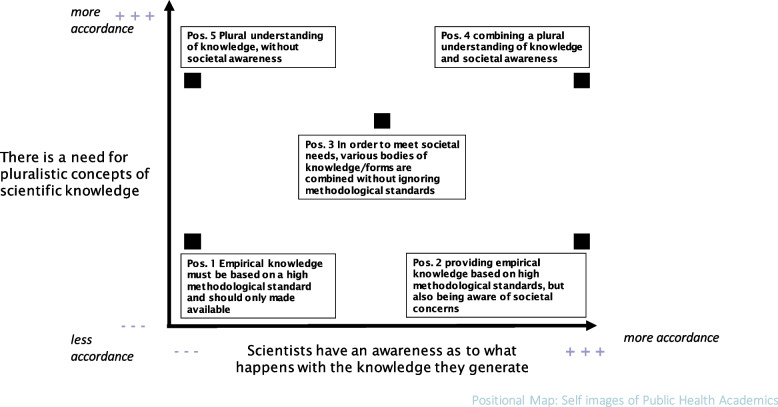
Positional map: Positions taken on knowledge and politics

SA as a form of Grounded Theory (GT) uses the methods of GT, but differs from it especially in terms of its research focus. While GT focuses on the social interactions of agents, the SA illuminates situations from different angles (Clarke 2021, p. 224–225). In this project, we used SA to view the situation of PH in the area of tension between science and politics. By mapping the arena of PH in Germany, we visualized localizations and relations among the collective actors, institutions and social worlds involved (Fig. 1). In the research process different positions regarding knowledge production and societal awareness were voiced which led to positional maps such as Fig. 2. These positions revealed aspects of the relationship between PH and science and led us to the development of the typology of self-images of PH academics presented in this article. As the mapping data are extensive and we consider the self-images to be highly relevant, there is only one situational map of the social arena and one positional map shown as an example in this article.

## Data collection and data processing

We collected 36 published documents (statements, position papers, fact sheets, open letters) published by the German Society of Public Health (DGPH), the German Society for Social Medicine and Prevention (DGSMP), the German Society for Epidemiology (DGEpi), the German Society for Medical Sociology (DGMS) and the German Society for Medical Informatics, Biometry and Epidemiology (GMDS) in 2020–2021. These focused on major issues that arose during the pandemic (e.g. first and second (partial) lockdowns and school closings, vaccination campaigns and testing regimes). We also considered selected documents from the German Public Health Competence Network on COVID-19. Additionally, we conducted 21 reflexive online interviews with academics from the PH discipline by using a thematic guide described in the study protocol [28].

### Sample

In 2022, we recruited interview participants, listed in Table 1, via scientific networks, professional contacts and snowballing [27]. The interviewed PH academics were from institutions in different regions of Germany. The highest academic grade held respectively was a professorship at a German university for 17 of them, a PhD for two individuals and a master’s-level academic degree for another two. The sample not only reflected the heterogeneity of PH academia but also regarding other characteristics (e.g. age, gender, degree of seniority). All participants were interviewed once, and repeat-interviews were not carried out. Sampling continued until theoretical saturation was reached. We assumed saturation when no further aspects of the understanding of the position of PH toward politics emerged (Morse 2015). At that point, the interview partners also started referencing academics from the German PH sector, whom we had already interviewed.

**Table 1:** Disciplines and affiliations of the interview participants

No	ID	Gender	Discipline	Affiliation	Academic position
1	Gm	m	public health	university	professorship
2	Cgh	f	public health, social medicine	university	professorship
3	Ds	m	epidemiology	institution	PhD
4	Fg	m	medical sociology	university	professorship
5	Wf	f	medical ethics	university	professorship
6	Hp	f	public health	university	professorship
7	Zm	f	statistic methodology	university	PhD
8	Pk	m	medical sociology	university	professorship
9	Te	f	epidemiology	institution	professorship
10	Br	f	health science	institution	master-level
11	Ls	m	public health	university	professorship
12	Yb	m	health research, medical sociology	university	professorship
13	Jd	f	public health, medicine	university	professorship
14	Uj	m	epidemiology, public health	university	professorship
15	Sö	f	epidemiology	university	professorship
16	Rn	f	medical sociology	institution	professorship
17	Mk	m	public health	university of applied science	professorship
18	Kj	m	public health	university of applied science	professorship
19	Ip	m	public health	institution	master-level
20	Vh	f	public health	university of applied science	professorship
21	Ot	f	nursing science, epidemiology	university	professorship

We received written informed consent from the participants. Since we were aware that the interviewees knew each other for the most part, we were particularly careful in protecting personal data by following a strict data privacy concept. Ethical approval was obtained from the ethics committee of Otto von Guericke University Magdeburg (no. 196–21).

### Data analysis

To analyse our data, we combined analytic approaches from GT and SA [8, 10]. The data were transcribed verbatim and subjected to respondent validation. Subsequently, the research process was guided by memoing and mapping the situation, coding, recoding the data, and defining categories, which eventually led us to develop a typology. We used categories for mapping procedures. Starting with (1) situational mapping to gather all the elements of the situation under research found in the data, we further structured the data with (2) ordered mapping. We then created different social world/arena maps on negotiated issues in the situation [9] (p. 104). After, we (3) focused on the orientations and values of PH academics in coding, addressing the crisis situation as ‘key elements’ of the discursive data [9] (p. 206). Finally, (4) we developed five types of self-images of PH academics presenting similarities and ambiguities in their orientations during the pandemic. As stated in our research protocol, the empirical results were theoretically reflected to enhance the interpretation of the data regarding the relationship between evidence and politics (or science and politics). We used philosophical concepts pertaining to the relation between politics and science as well as the role of scientist as a heuristic, to clarify and determine associated values. In concrete terms we refer to Habermas’ definitions of technocratic, decisionist and pragmatist understandings of the relation between politics and science as well as to Manson’s concept of the ‘epistemic restraint’ and Horkheimer’s model of the ‘critical theorist’ [18, 20, 25]. We elaborate on these further in the discussion section of this paper. The interdisciplinary research team recurrently discussed the findings.

## Findings: Social Arena maps, Positional maps, self-images in the PH community and general findings

We incorporated the results of situational and ordered mapping into a social arena map as shown below in Fig. 1.

Subsequently we used positional maps to visualize positions taken in the discourse around the pandemic situation. A positional map depicting positions regarding methodologies of knowledge production and societal awareness is shown in Fig. 2. In the process we observed that the positions that supported societal awareness in the creation of knowledge seemed to be more sensitive to political interventions while other positions merely focused on generating knowledge.

After realising this we categorized aspects of the relationship between science and politics. Supported by the coding of the interviews, we then developed a typology of self-images. We considered self-images to be most relevant and therefore mainly focused on their analysis and discussion. As listed below in Table 2, five types of self-images show aspects regarding what PH academics define as relevant for their discipline facing the COVID-19 pandemic. We classified the self-image types by orientation and associated values.

**Table 2:** Typology of self-images of public health academics during the pandemic

Type	Orientation	Associated values
scientific study supplier	specialization “I do high quality research.”	scientific integrity/soundness, neutrality, unbiased independency
expert facing political issues	contribution “I provide data and make findings usable for policy-makers.”	exchange and cooperation, dialogue, dependant on social and political needs
restrained scholar	redirection/reverse “I withdraw and concentrate on other subjects.”	caution, family responsibility, self-care reticence, practicality
public informer	information/transfer “I communicate with the public and inform about health issues.”	transparency, transfer of knowledge, public as essential for democracy
changemaker	structural critique “I work on transformation.”	equal life conditions, health as political issue, politization (of science), involvement

Through a comparative analysis, we identified overlaps and contradictions between empirical materials, which captures the variety of types [22]. A type is not assigned to individuals or institutions but represents the diversity of self-images in PH. As the self-images emerged from coding and evaluating key elements of all interviews, they are not tied to the interviewees per se. One PH researcher would therefore not necessarily embody one certain type. Rather, the researcher can represent aspects of more than one type simultaneously (and even aspects of seemingly mutually exclusive types). Similarly, the aspects of the type(s) one PH researcher might represent were subject to change as the pandemic progressed. For instance, some researchers tried to engage in discourse at the start of the pandemic, representing elements of the scientific study supplier. However, later they withdrew, thus embodying more the restrained scholar. Furthermore, researchers may have prioritized different aspects of their self-image as academics at certain times throughout the pandemic. Types are then not assigned to individuals or institutions but represent the diversity of self-images in PH. While the values and orientations of single researchers were subject to change during the pandemic, our typology of the self-images should serve as a (more or less) fixed scheme. This scheme can be used to observe and reflect such changes. Last, it is worth mentioning that the self-image that a person in their role as scientist embodies can still be influenced by and associated with personal values such as family responsibility. Whether gender, age, educational background or other factors such as race play a role in the type of self-image, could also be considered. This was, however, not analysed in our study.

In the subsequent sections, we present the types following the order of the table above.

### The scientific study supplier

This type of self-image is characterised by a strict separation of science, politics and society as specialized worlds. It is the task of a PH academic to maintain these distinctions, especially during a crisis. Based on this self-image the researcher should focus on generating evidence according to the highest scientific standards. Hence, these researchers see the preservation of scientific integrity as a central value guiding their work.

Well, what we can do is […] to organize data, to evaluate studies and to present the whole thing with as high a methodological standard as possible [...] primarily I see my task in doing research based on a good methodological level. *(Fg, 4)*

In the interview quote, Fg recurs to scientific principles that must be followed. From this perspective, PH science must collect and organize. Furthermore, it has to provide an overview of the generated data in the current and future pandemic situations.

[W]hat I, as a (laughs) private person, would expect from science [...] simply that yes, to be unbiased and- but also at the same time clear analyses actually come together, which then also offer a clear orientation for where or for what measures are useful […]. From my point of view, this is how science should act. *(Ls, 16)*

Ls constructs clarity in opposition to confusion (‘unbiased’). Science, in his understanding, thus establishes order (‘orientation’) in a chaotic situation and builds up stability and reliability. Ls does not address concrete recipients of his scientific activities, but refers to evaluating the usefulness of political pandemic control measures.

In general, this type is based on a more service-oriented self-understanding. This mainly amounts to providing high quality data that are appropriately and efficiently processed for prevention and health promotion.

### The expert facing political issues

The second type of self-image perceives PH academics as contributors to policy-making. This contribution intertwines with associated values of exchange and cooperation as well as dialogue dependent on social and political needs. The statement of the Competence Network Public Health on COVID-19, which was formed during the pandemic, defines how knowledge from research should be used:

The aim is to provide rapid and flexible interdisciplinary expertise on COVID-19 for current discussion and decision-making. *(Statement of Public Health Competence Network on COVID-19, n.d., #38)*

In addition to a rapid synthesis of knowledge, the authors of the statement see their purpose to help policy-making decisions by supplying interdisciplinary expertise. The self-image of PH is characterised by working for the benefit of political needs addressed by decision-makers, especially in a crisis. Ds, for example, works in a federal research institute, which is primarily responsible for health reporting. In contrast to the Competence Network, he had to address orders articulated by policy-makers during the pandemic:

[...] but of course we also get more work orders from the political side, which we then [...] have to serve and where we are then just no longer completely independent in what we do [...]. *(Ds, 21)*

According to Ds’ experience, policy-makers influence research topics adopted by the scientific community, blocking PH scientists from other subjects, especially in a crisis. Uj sees his role as a PH scientist in providing political advice voluntarily. During the pandemic, he remembered PH values that were central to his self-image:

[...] the guiding principle at Johns Hopkins [university] is to protect […] health, [...] saving lives, millions at a time. […] and that impressed me […] It's actually a guiding principle of public health, and I think it fits generally to applied health sciences. And that's why I think (...) in our disciplines, […] it's about benefit, and that's why I think it must be an integral part to also be politically active in advisory processes. *(Uj, 12)*

From Uj's perspective, not only PH scientists, but also academics working in applied sciences are responsible for promoting population health through advisory activities.

The self-image of PH in this type is to inform political decision-making by collaborating with politicians.

### The restrained scholar

Generally, the restrained scholar is characterized by unsuccessful involvement in the scientific discourse and a consequential withdrawal and shift in focus. First, we observed a decrease in publications from scientific societies from mid-2021 onward. Second, some academics we interviewed reported a withdrawal after an initial flurry activity. Wf described:

In the beginning, I had this active involvement and “science is needed here” and “we can do something” and so on. And it has changed to the extent that I thought no, I don't really have a role in this right now. Because it is not demanded. Not needed, so that I then concentrated again on my core business […]. I have taken over this professorship […] I had to carry my children through home schooling and so on. *(Wf, 18)*

After the first phase of the pandemic, Wf had to prioritize other responsibilities and did not concentrate on sharing her expertise to provide information about COVID-19. In the interview, she also talks about her experience of ‘frustration’, ‘disillusionment’ and ‘exhaustion’ regarding what she perceived as policy-makers' lack of interest in PH. Her efforts to explain the pandemic in the media from a PH perspective took much time and effort, so she decided to withdraw during the pandemic.

Another case of a restrained scholar is Sö. During the initial phase of the pandemic, she observed the progress of PH network initiatives and concluded to discontinue her participation.

[...] I personally felt that these structures became rather quickly so large and complex that fast reactions were no longer possible, which then led me personally to withdraw a bit. *(Sö, 37)*

The size of the network and the dynamics of who was involved led her to focus on subjects that received less attention. She explained: “I had the feeling: Okay, you have now made your contribution. You have voiced your concerns” (Sö, 49). In the way she perceived herself, it was part of her role to fulfil a function in a temporary situation as an authority (to warn), acknowledged and appreciated by her PH community.

In contrast, Zm gives a different reason why she felt restrained during the pandemic:

[…] as a statistician, especially in the beginning, […], I was very critical of either what was in the press or what [...] colleagues published and considered themselves to be experts. I have personally not participated in an active contribution. [...] I have two children and for me my personal situation was the important one and I actually did not feel objective. […] I had the feeling of being personally very affected by political decisions. And that's why COVID was actually a huge emotional issue for me, […] I don't want to deal with it professionally either. *(Zm, 4)*

First, her disciplinary affiliation as a statistician in a PH institute held her back from more activity on the subject. The critical stance she took toward her colleagues at the beginning of the pandemic, was followed by becoming too intertwined with her ‘private’ experiences. Being personally affected prevented her from scientifically focusing on COVID-19 and actively participating in the discourse on the pandemic. She refers to similar values as the scientific study supplier (type 1), by critically questioning herself to remain objective.

According to our data, there are two different ways to retreat: Wf and Zm mention personal issues (e.g. care) as reasons to restrain themselves from research as such. In opposition, Sö did not restrain herself from her scientific work but only changed her focus to non-Covid-19 related research topics. The withdrawal of PH academia from COVID-19 initiatives was due to frustration about a lack of attention from politicians, a shift of focus to other research topics or personal afflictedness. The most important values associated with this type are caution, family responsibility, reticence and self-care.

### The public informer

The data to support the self-image of the public informer were limited. However, our interviews with almost all PH academics revealed that communicating knowledge to the public during the pandemic marked a crucial element of their discipline. Gm explains:

If you come from the public health field, you know how important communication is. [...] We do not have many more weapons, so to speak. In the meantime, we still have vaccination. But all the other things, masks and so on, that's all based on people doing it. *(Gm, 26)*

For Gm, it was a “civic duty” to communicate research findings and information to the public. Communication was considered an important tool for pandemic management. Apart from Gm, a minority of interview participants described how they came to address the public on COVID-19 in various media formats. Some academics were in close contact with the media, whereas others removed themselves completely from the media after experiencing an overwhelming number of media requests (see type of the restrained scholar).

Most PH academics observed a lack of public representation of their discipline, e.g. by a particular individual. However, Ls takes a critical stance on this:

Public health is a multi-layered and multi-voiced science. Of course, it is ideal to have a public face […]. But it would also have made me feel a bit alienated for public health. *(Ls, 22)*

Scientific societies in the field of PH considered it to be their primary task to make their knowledge available for the scientific community and to politicians but not to the public. For example, the Competence Network Public Health on COVID-19 notes that the information they provided was “primarily aimed at authorities, institutions and political decision-makers” (#36). Another voice from the PH community perceived a conflict between the roles of scientists and public informers:

We have tried repeatedly to present our results in a way that is understandable to lay people [...]. However, in the end, no one can expect that from science [...].Well, I could imagine doing something like that, but I cannot be a scientist and do science communication or journalism or campaign planning. It does not fit together. *(Rn, 37)*

Thus, while PH academics exercise caution due to an expected role conflict, they point out a lack of role models in their discipline for communication with the public. Jd found inspiration in a domain beyond PH:

This virologist [...] She explained to the public on how the vaccination was actually developed and so on. I thought that was very good and then in an interview she said she was asked about all the talk shows and where all her colleagues are hosted and she just said very cool: Who needs talk shows in this situation? And yes. So she was a bit of a role model to me. *(Jd, 19)*

Both data sources, interviews and documents, reflect a defensive and careful approach to the media. Pk, for example, noted that scientists should be careful when talking about research findings in public (“you must consider for what purpose”, Pk, 3). The German Society for Medical Informatics, Biometry and Epidemiology (GMDS) noted a related problem that arose in the pandemic:

In a liberal democratic society, critical reflection by the press is therefore all the more important. However, the GMDS strongly rejects any untruthful insinuation of misinformation and demands the necessary personal respect when dealing with scientists and researchers. *(GMDS, Statement on handling of reporting on SARS-CoV-2, 25.05.2020, #8)*

In contrast, PH academics perceived it as part of their job to inform and, if necessary, explain scientific facts to the public. This is essential to maintaining democratic structures. At the same time, they ascribed the main responsibility for health communication during the pandemic to politicians. The majority of the PH academics we interviewed, parallel to the selected documents we analyzed, emphasized the values of transparency and transfer of knowledge, and criticized shortcomings in the pandemic by governmental institutions responsible for health communication in Germany.

### The changemaker

The fifth type of self-image of PH academics during the pandemic is the changemaker, who aims to promote social transformation. This type is a recurring theme of our data, however, it depends largely on the institutional and professional context of the actors. The changemaker is characterized by voicing critique, as represented by Te:

I am still driven by the unequal distribution of health opportunities and this is, in fact, not given by nature, but is a result of constructed social inequalities. *(Te, 21)*

This self-image of PH academics focuses on working to overcome inequities in society and to support equality and strengthen democratic structures (“fixing social inequality is also an important value to me”, Rn, 25). To this end, it is important to consider “collateral damage right from the start” of the pandemic (WF, 16). Moreover, PH academics in this type are critical of technical terms and categorizations. For example, the German Scientific Society for Public Health suggests:

[…] in order to avoid stigmatization of so-called “vulnerable” groups, the “proportionate universalism” approach proposed by Michael Marmot ought to be adopted. *(DGPH statement addressed to the CDU party in the Thuringian state parliament “To strengthen public health”, 09.09.2021, #30)*

The authors seek solutions and propose the use of sensitive language to address specific population groups. The changemaker draws attention to the health impact of inequality and, furthermore, problematizes the role of science in cementing unequal societal structures. Their motivation seems to be fuelled by idealism.

For example, Ip remarks: “We always dream of health in all policies. That is our guiding star, so to speak, the WHO approach, health in all policies” (Ip, 13).

This core value of PH is also reflected in the position of Rn. She believes that a “classical understanding of public health” includes focusing on “not only social inequality, but also real solidarity”.“[S]uch a crisis also means, I help other people in some way” (Rn, 27). This perspective was echoed by PH academics who, for instance, volunteered in vaccination campaigns.

### General findings

Due to our methodology of GT and SA we uncovered different relationships in the worlds and subworlds. Across the data found in the interviews, we observed a general feeling of not being heard enough, being marginalized by politics and having negative media experience. We found reluctance to communicate to the public sphere and with the media. Some study participants withdrew from the public eye because of negative experiences in the past. Others observed how their colleagues were seemingly exploited by the media. PH academics criticize that social factors of health were not taken into account at all or only at a later point in time by politicians. This feeling of marginalization was not directly associated with a certain type. Moreover, for some PH academics, juggling academic and personal demands such as care work presented unique challenges that shaped their self-image. Furthermore, the results confirm that PH as a discipline aims to challenge a biomedical bias regarding health. For instance, the expert facing political issues and the changemaker incorporate a critical perspective on a single biomedical understanding of health. They vote to also consider social factors of health. The focus of politics and the public media on the biomedical sciences also led some scholars to retreat from research on Covid-19.

## Discussion

Our study identified five different types of self-images in PH academia during the pandemic in Germany with different orientations and associated values: On the one hand, the study supplier makes a scientific contribution with tailored research. On the other hand, the expert and the public informer provide research findings for politicians, PH practitioners and, on rare occasions, the public. How sparsely the public was informed by both types contradicts the PH principle of supporting the dissemination of PH knowledge consistently occuring in our data. Our findings confirm first, the close connections and great degree of engagement of German PH scientists during the critical phases of the pandemic. Second, the data revealed a perceived lack of representation in political advisory boards and the media [12, 17, 30].

Aspects of the self-images can also be found in the aforementioned theoretical concepts by Habermas, Manson and Horkheimer. The scientific study supplier for instance corresponds (partly) with Habermas’ description of the technocratic understanding of science. Here, the scientists’ role is to ‘rationalize’ political questions through elaborate methodological approaches. They do so without taking into account social needs, public opinions or questions of political transformation. The expert facing political issues corresponds partly with Habermas’ description of a decisionist understanding of the relation between science and politics. Accordingly, scientists answer to the needs of politicians by providing a foundation and strategic repertoire for decision-making. Habermas himself argues for a pragmatist understanding of the relation between science, policy and the public. From his viewpoint, the public plays an important role as a liberal-democratic mediator between scientific evidence(s) and political decision-making based on values. In this light, scientists have an ethical responsibility to participate in public discourses about e.g. practical implications of scientific recommendations. This pragmatist understanding is embodied by the type of the public informer. The restrained scholar’s self-image corresponds with the attitude and practice of ‘epistemic restraint’, as introduced by Manson [25]. By drawing on philosophical critique of the virtue of curiosity, Manson argues that restraint can be an important virtue, as epistemic pursuits have (e.g. ‘private’) opportunity costs and pose risks and burdens. Restraint can increase integrity, as it is often associated with humbleness, self-critique and authenticity. The self-image of the scientific changemaker partly corresponds with Horkheimer’s [20] concept of the ‘critical theorist’ in contrast to traditional theoretical and scientific work and writing. The critical theorist traces unrealized emancipatory potential (e.g. conditions for health equality) back to the structural roots of society. The critical theorist's understanding of science is political which involves the idea that science can and should contribute to beneficial social transformation.

Apart from the typology of self-images, we encountered a general discomfort in the academic PH community facing decisions made by policy-makers during the pandemic such as the closing of schools over long periods of time. These decisions were criticized and questioned regarding their justification. Furthermore, pandemic policy has been assumed to largely ignore and even promote social inequalities, which resonates with several PH authors [21, 23, 33]. For instance, in our data, the restrained scholar is a type mostly embodied by women who restrained themselves from their scientific research possibly due to the larger burden of care work. This seems plausible as studies show that Covid-19 measures in Germany partially reproduced patriarchal power structures. This points to a gendered dimensions of inequality that impacted well-being and academic careers [3, 4, 11]. Whether the restraint scholar is actually dependent on gender needs to be analysed in future research.

The feeling of not being heard is also connected to negative media experience and a mention of biomedical bias. The restrained scholar proves to be distinctive in this case as the feeling of marginalization, of not being heard, was precisely the reason for withdrawal from the discourse in some cases. Being marginalized also applies to the restrained scholar in a different way, since their position is not represented in our positional map regarding positions taken in the discourse. The restrained scholar is not being represented although some PH researchers embody this type.

Self-images of PH academics reflect an interdisciplinary discourse on the normative foundations of their discipline. The self-image of the changemaker appears to be relevant for PH. This seems to be all the more the case with regard to future crises and the role of PH within the democratic social order [32], as the changemaker pursues political intentions, expanding the perspectives of biomedical disciplines. Changemakers have a politicized understanding of scientific practice, as they not only criticize political decision-making, but also tackle the structural level of unequal health and living conditions. In the current discourse, Speed and McLaren discuss a social democratic model of PH to counter neoliberal individualization of PH [32]. Future research may explore whether this self-image is also shared by other health disciplines. However, the typology of self-images is not meant to entail normative assumptions. Researchers of PH and other disciplines can rather decide to prioritize certain values at times and embody certain self-images. The heterogeneity of orientations, values and priorities in particular could be seen as beneficial to an interdisciplinary field such as PH.

### Limitations and strengths of the study

In opposition to the research plan set out in the study protocol, we did not make full use of the mapping tools provided by the [28]. In addition to social world/arena mapping and positional mapping, we primarily performed GT-coding to generate the typology, while mapping procedures served as analytical tools [26]. By combining them with GT, we were able to depict variations and contradictions in self-images within the PH community. Our study shows only a segment of the discipline, because we focused on PH academia in Germany. It is therefore difficult to generalize our findings and transfer them to other subdisciplines or even other disciplines. To differentiate values, that are associated with self-images, we used concepts from social theory and philosophy as points of reference. Therefore, the typology also makes a theoretical contribution.

In this article, we based the typology of self-images on the interviews of individual researchers and the statements of organizations. The term “terminology” entails an abstract, formal, fixed concept that can be derived from empirical analysis. The types do not necessarily exist as such in reality. However, individuals can embody aspects of these types, which can be subject to change over time. Publications and statements by organizations can hint at collective positions and discursive discussions on the topic. These statements can therefore be seen as a condensed voice of the collective. Whether single organizations, research societies or researchers can be identified with the types developed in this paper, was not part of our research goals but would be an interesting inquiry for further research.

As our study was conducted in Germany, our results cannot be directly compared and applied to the international PH sector. In contrast to the Anglo-Saxon model, the German PH system appears to be largely fragmented and is administered by various federal and communal agents, as shown in Fig. 1. Simultaneously, a large number of federal and local, governmental and non-governmental PH institutions, unions and health insurers facilitate PH-research, PH-services and policy-making. However, the PH-lobbying group Future Forum Public Health (Zukunftsforum Public Health), among others, has published a detailed report on how PH should ideally be administered in Germany to strengthen the PH sector and build pandemic preparedness [37].

Crisis management was primarily initiated by political authorities. Academics working in policy-related institutions and participating in crisis management were therefore significantly curtailed in their capacity for independent research decisions. We did not consider power structures in the practice of PH services in our study. Covid-19 has brought to light inequality structures within society influencing which groups were most affected by Covid-19 and whose interests are being focused on in the undertaking of measures [24]. Power structures within PH academia are often not investigated or reflected upon. For instance, questions such as which subdisciplines of PH receive public and intradisciplinary attention or whether female researchers are more likely to restrain themselves as scholars due to care burden or structural disadvantages among many other inquiries, should be addressed in future research.

Furthermore, potential biases and power structures within the research group should be mentioned. The research group consisted of two male professors and three female and one male research fellow all of whom are white. Although the hierarchies are flat, the empirical analysis conducted by two female researchers was supervised by a male professor. We can despite our best efforts not foreclose, that this might have influenced the ways the research was conducted and interpreted.

## Conclusion

Our findings show that PH academia represents a heterogeneous image of the discipline of PH that is not always in line with established PH values (e.g. WHO charter). Making orientations and values associated with the varying self-images explicit appears important for enabling inter- and transdisciplinary work in the future and managing expectations in interactions between PH and the political arena. Awareness of the different self-images in PH could promote discourse on the future development of the discipline and encourage reflection on the role of PH in society.

## Data Availability

The data was collected in a primary study as part of the project and is stored on a protected server with access for project staff only.
